# Calcium- and Integrin-Binding Protein 1 Regulates Endomitosis and Its Interaction with Polo-Like Kinase 3 Is Enhanced in Endomitotic Dami Cells

**DOI:** 10.1371/journal.pone.0014513

**Published:** 2011-01-14

**Authors:** John C. Kostyak, Ulhas P. Naik

**Affiliations:** 1 Department of Biological Sciences, University of Delaware, Newark, Delaware, United States of America; 2 Department of Chemical Engineering, University of Delaware, Newark, Delaware, United States of America; 3 Delaware Biotechnology Institute, University of Delaware, Newark, Delaware, United States of America; 4 Department of Chemistry and Biochemistry, University of Delaware, Newark, Delaware, United States of America; 5 Delaware Cardiovascular Research Center, University of Delaware, Newark, Delaware, United States of America; Cleveland Clinic, United States of America

## Abstract

Endomitosis is a form of mitosis in which both karyokinesis and cytokinesis are interrupted and is a hallmark of megakaryocyte differentiation. Very little is known about how such a dramatic alteration of the cell cycle in a physiological setting is achieved. Thrombopoietin-induced signaling is essential for induction of endomitosis. Here we show that calcium- and integrin-binding protein 1 (CIB1), a known regulator of platelet integrin α_IIb_β_3_ outside-in signaling, regulates endomitosis. We observed that CIB1 expression is increased in primary mouse megakaryocytes compared to mononuclear bone marrow cells as determined by Western blot analysis. Following PMA treatment of Dami cells, a megakaryoblastic cell line, we found that CIB1 protein expression increased concomitant with cell ploidy. Overexpression of CIB1 in Dami cells resulted in multilobated nuclei and led to increased time for a cell to complete cytokinesis as well as increased incidence of furrow regression as observed by time-lapse microscopy. Additionally, we found that surface expression of integrin α_IIb_β_3,_ an important megakaryocyte marker, was enhanced in CIB1 overexpressing cells as determined by flow cytometry. Furthermore, PMA treatment of CIB1 overexpressing cells led to increased ploidy compared to PMA treated control cells. Interestingly, expression of Polo-like kinase 3 (Plk3), an established CIB1-interacting protein and a key regulator of the mitotic process, decreased upon PMA treatment of Dami cells. Furthermore, PMA treatment augmented the interaction between CIB1 and Plk3, which depended on the duration of treatment. These data suggest that CIB1 is involved in regulating endomitosis, perhaps through its interaction with Plk3.

## Introduction

Megakaryocytes are large, polyploid cells that undergo a unique form of mitosis known as endomitosis. This physiological process allows the megakaryocyte to expand its volume and intracellular contents necessary to meet the demands of its ultimate function; platelet production. Endomitosis occurs due to an altered cell cycle in which cytokinesis is bypassed resulting in a cell with double the original DNA content. Repeated rounds of endomitosis allow the cell to become highly polyploid. Furthermore, the amount of platelets one megakaryocyte can produce is directly related to the number of endomitotic cycles that megakaryocyte undergoes [1]. It was recently illustrated that early megakaryocytes, such as dipolar megakaryocytes, form a furrow which later regresses [2]. Whereas, more mature megakaryocytes may not form a furrow at all [3]. Although, many proteins have been implicated in the regulation of endomitosis, the exact mechanism remains elusive.

Polo-like kinases are a group of evolutionarily conserved serine/threonine kinases, of which 5 are identified in mammals (Plk1, Plk2, Plk3, Plk4, and Plk5) and have been implicated in cell cycle regulation [4], [5], [6], [7], [8]. In particular, several functions relating to mitosis have been attributed to Plk3 (also termed Fnk) [9], [10], [11], [12]. Polo-like kinases are characterized by a carboxy-terminal polo-box domain that is necessary for subcellular localization [13], [14], [15]. In fact, when ectopically expressed, the polo-box domain of Plk3 localizes to the centrosomes, spindle poles, and midbody and eventually causes mitotic arrest and apoptosis [13]. Additionally, ectopic expression of both a constitutively active and a kinase dead Plk3 caused G(2)/M arrest and apoptosis [9]. Also, expression of a constitutively active Plk3 in lung carcinoma cells promoted an elongated and unsevered midbody [9]. Furthermore, Plk3 is able to phosphorylate Cdc25C, which allows Cdc25C to enter the nucleus and promote mitosis [12]. Plk3 is also integral to the DNA damage response as it phosphorylates both Chk2 and p53 [11], [16]. Thus, Plk3 activity and localization may dictate cell cycle progression and perhaps endomitosis.

Calcium- and integrin-binding protein 1 (CIB1) was originally identified as a binding partner of the megakaryocyte lineage-specific integrin α_IIb_β_3_ [17]. Since that discovery, CIB1 has been shown to bind and regulate a variety of signaling proteins [8], [18], [19], [20]. Although, CIB1 has been recognized as an important platelet regulatory protein, very little is known regarding its role in megakaryocytes. Recently, we demonstrated that CIB1 not only binds, but also inhibits Plk3 kinase activity [21]. Given that both CIB1 and Plk3 are expressed in megakaryocytes, they interact, and Plk3 is implicated in several processes related to mitosis, it is reasonable to predict that CIB1 and Plk3 have a role in endomitosis.

In this report, we demonstrate that CIB1 is involved in endomitosis and that it may enhance cell ploidy through an interaction with Plk3. Here we show that CIB1 protein expression increases upon PMA treatment of Dami cells. This finding is substantiated by our observation of increased expression of CIB1 in megakaryocytes treated with thrombopoietin (TPO) when compared to bone marrow mononuclear cells. We also show that overexpression of CIB1 causes increased PMA-dependent ploidy, increased α_IIb_β_3_ surface expression, and an increased tendency of furrow regression. Furthermore, knockdown of CIB1 using a RNAi approach in Dami cells decreased PMA-induced ploidy. Additionally, we show that CIB1 interacts with Plk3 in Dami cells and that the interaction is enhanced in the presence of PMA. These data strongly suggest that CIB1 is involved in the regulation of endomitosis.

## Materials and Methods

### Antibodies and reagents

Unless otherwise stated, all reagents were purchased from Sigma-Aldrich (St. Louis, MO). Production of UN7.79 (CIB1) was described previously [17]. Polyclonal antibodies against CIB1 and HSC-70 were purchased from Santa Cruz Biotechnology (Santa Cruz, CA). A monoclonal antibody against the integrin α_IIb_β_3_ (10E5) was a generous gift from Dr. Barry Coller (Rockefeller University, New York, NY). The monoclonal antibody against Plk3 was purchased from Becton-Dickinson (Franklin Lakes, NJ).

### Cell culture and transfection

Dami (human megakaryoblastic) cells were obtained from the American Type Culture Collection (Manassas, VA). Cells were maintained in Iscoves Modified Dulbeccos Medium (IMDM) supplemented with 10% heat-inactivated fetal bovine serum, 100 U/mL penicillin, and 100 µg/mL streptomycin (Invitrogen, Carlsbad, CA). CIB1 cDNA was cloned into the expression vector pEGFP-N1 and cells were transfected using lipofectamine 2000 (Invitrogen) according to the manufacturers instructions and as previously described [18]. Control transfections were carried out using empty vector. Geneticin (G418) was added to the cuture media 48 hours following transfection at a concentration of 500 µg/mL for selection. The resistant colonies were isolated and maintained in culture medium containing 300 µg/mL G418. Transfection efficiency was assessed by Western blot. Three CIB1-EGFP overexpressing (CIB1oe) and control (mock) clones were used for these studies. CIB1 and Plk3 shRNA's have been previously described [22]. Transfections were carried out using Superfect reagent (Qiagen, Valencia, CA) as per the manufacturer's instructions. The population of transfected cells was enriched using 10 µg/mL puromycin. CIB1 depleted cells (CIB1^sh^), Plk3 depleted cells (Plk3^sh^), and mock transfected cells (Mock^sh^) were treated with 100 nM PMA for 3 days prior to ploidy analysis.

### Cell viability and proliferation

For proliferation experiments, 10^4^ cells were plated in growth media and counted via a hemocytometer 72, 144, and 196 hours after plating. Concurrently, cells were loaded with 0.2% trypan blue to identify the cells which were not viable. Cells that absorbed the dye were considered not viable and quantified [23].

### Time-lapse microscopy

CIB1oe or mock Dami cells (5×10^5^) were plated in an 8-well dish and supplemented with selection media. Cells were incubated in a controlled environmental chamber at 37°C and 5% CO_2_. Images were captured every 2 minutes for 24 hours using a Zeiss Axiovert 2000 microscope with a Hoffman module (Carl Zeiss Inc, Thronwood, NY). Time-lapse videos were produced using Axiovision software (Zeiss). Cytokinesis time was defined as the point a furrow was visualized to the point at which two distinct cells were visible. A total of 18 videos were produced using 3 CIB1oe clones and 2 mock clones.

### Surface expression of the integrin α_IIb_β_3_

To determine the integrin α_IIb_β_3_ surface expression, 10^6^ CIB1oe or mock cells were labeled with 10E5 antibody (1∶5000) or isotype-specific IgG (Santa Cruz Biotechnology) for 45 minutes at 4°C. Cells were then incubated with a FITC-tagged anti-mouse secondary antibody (1∶250) (Jackson Laboratory, Bar Harbor, ME) for 45 minutes at 4°C and then fixed in 4% paraformaldehyde for 10 minutes at room temperature (RT). Cells were then analyzed using a FACScalibur (Becton Dickinson) flow cytometer. A total of 20,000 cells were counted for each sample, in triplicate.

### Ploidy analysis

CIB1oe and mock cells were serum starved overnight in IMDM containing 0.1% BSA. 10^5^ cells were then plated in 8-well dishes coated with 10 µg/mL fibronectin and allowed to spread. Following spreading, the cells were stained with Wright-Giemsa (Fisher Scientific, Pittsburgh, PA). DNA content was assessed as reported previously [24], with minor modifications. Briefly, 2×10^6^ CIB1oe or mock cells, either treated with 100 nM PMA or vehicle, were fixed in 0.5% formalin for 10 minutes at RT. Following washing, cells were permeabilized in 70% methanol for 1 hour at 4°C. The cells were then washed prior to addition of 1 mg/mL RNase A and incubated at 37°C for 30 minutes. The DNA was then labeled with 1 mg/mL propidium iodide and analyzed using a FACSaria with FACSdiva software (Becton Dickinson).

### Immunoprecipitation and Western blotting

Immunoprecipitation and Western blotting were performed as previously described [25]. Briefly, washed cells were lysed using ice cold NP-40 lysis buffer (1% NP-40, 150 mM NaCl, and 50 mM Tris-HCl pH 7.5 containing 10 µg/mL each of leupeptin and aprotinin, and 1 mM each of NaF and PMSF). Equal amounts of protein were mixed with Laemmli buffer, boiled for 5 minutes, and separated via SDS-PAGE. The proteins were then transferred to PVDF membrane, blocked with 5% non-fat dry milk in TBST, and incubated with primary antibodies against CIB1 (1∶1000) or Plk3 (1∶1000), overnight at 4°C. Following incubation with the appropriate HRP-conjugated secondary antibody, the bands were visualized by chemiluminescence with Lumiglo® substrate (New England Biolabs, Ipswich, MA). The blots were then stripped and reprobed to assess loading using anti-HSC70 (1∶20000). For immunoprecipitation experiments, lysates were precleared using protein-G Sepharose beads prior to incubation with anti-Plk3 antibody (1∶250) or isotype-matched IgG overnight at 4°C. Washed protein G-Sepharose beads were then added for 1 hour at RT to capture the immunocomplexes. Following washing in NP-40 lysis buffer, the samples were processed for Western blotting.

### Murine megakaryocyte isolation from bone marrow

Bone marrow was extracted from femurs and tibias of *Cib1^+/+^*, *Cib1^−/−^* [26]. Bone marrow cells were isolated by flushing the bone marrow with IMDM using a 200 µL pipette tip. Bone marrow isolates were then passed through a 22 gauge needle to create a single-cell suspension, followed by centrifugation at 340×g for 3 minutes. The cells were then washed with phosphate buffered saline (PBS) prior to centrifugation at the same speed and duration. The cells were resuspended in ACK buffer (0.15M NH_4_Cl, 10 mM KHCO_3_, 0.1 mM Na_2_EDTA pH 7.4) for 5 minutes at RT to lyse the red cells. Following washing in PBS, the remaining cells were plated in IMDM with 10% FBS, 100 U/mL penicillin and 100 µg/mL streptomycin, and supplemented with 50 ng/mL recombinant mouse TPO (Peprotech, Rocky Hill, NJ). The cells were then allowed to grow for 5 days prior to separation (megakaryocyte and mononuclear cells) using a discontinuous BSA gradient (0%, 1.5%, 3%) as described previously [27]. All animal protocols were approved by the University of Delaware Institutional Animal Care and Use Committee (A3773-01).

## Results

### CIB1 protein expression is elevated in polyploid cells

CIB1 was first discovered as a binding partner of platelet integrin α_IIb_β_3_ and has been shown to regulate outside-in signaling during platelet activation [17], [18], [28], [29]. While platelets do have the ability to synthesize proteins, the majority of their intracellular contents are donated by the megakaryocyte. Therefore, we sought to determine if CIB1 also has a function in megakaryopoiesis. We chose to use Dami cells, a commonly used model cell line for human megakaryocyte development that has been shown to attain a megakaryocyte-like phenotype upon Phorbol 12-myristate 13-acetate (PMA) treatment [30]. PMA is commonly used to elicit a megakaryocyte phenotype in certain cell lines of hematopoietic origin as it activates several proteins necessary for endomitosis [31], [32].

**Figure 1:**
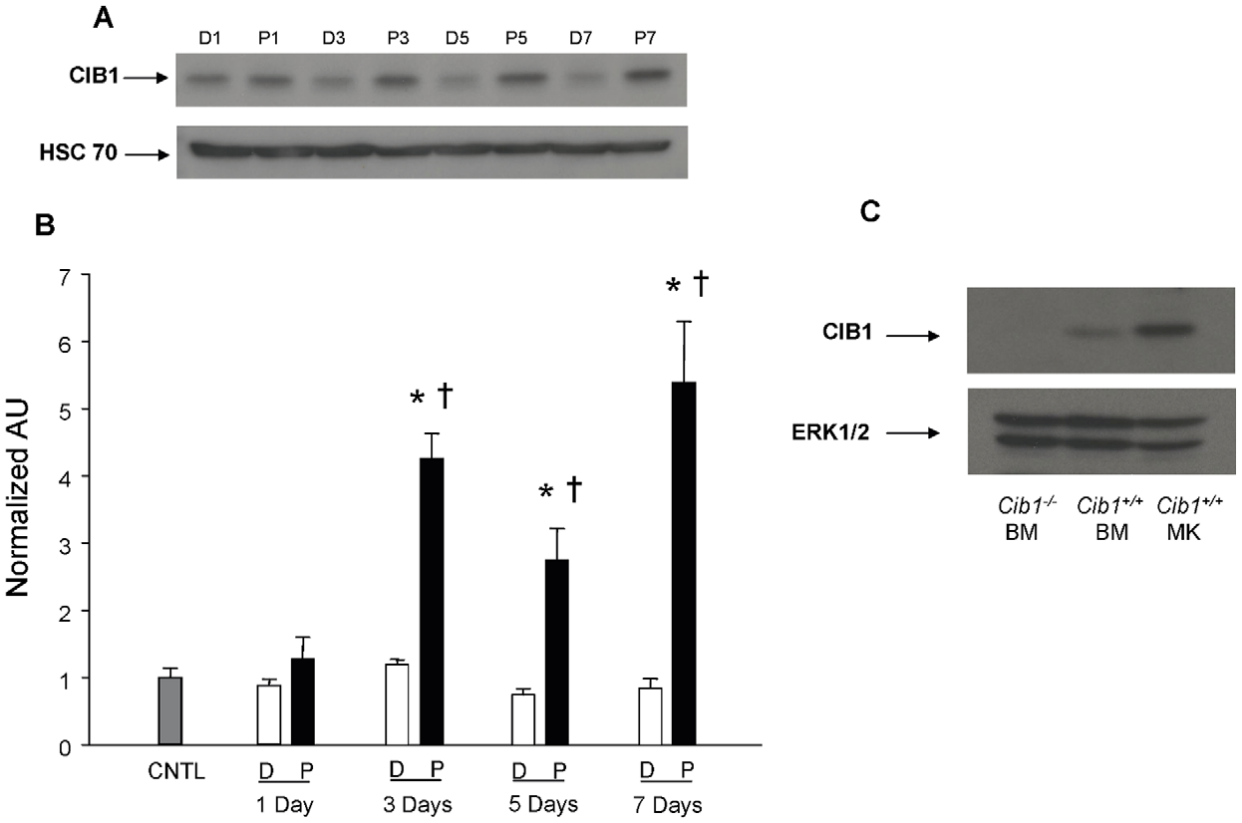
CIB1 protein expression is upregulated upon PMA treatment of Dami cells. A) Western blots of lysates from Dami cells treated with either 100 nM PMA (P) or vehicle control, DMSO (D), for 1, 3, 5, or 7 days in culture. B) Quantification of densitometric data obtained from (A) normalized to untreated control samples (CNTL). *P<0.05 vs CNTL; †P<0.05 vs corresponding DMSO value. C) Western blot of lysates from bone marrow cells isolated from *Cib1^+/+^* and *Cib1^−/−^* mice and megakaryocytes purified from cultured *Cib1^+/+^* bone marrow cells in the presence of 50 ng/mL TPO.

We first determined if CIB1 protein expression is altered upon treatment of Dami cells with PMA. As expected, DNA content (N) of Dami cells increased with increased exposure to PMA as indicated by flow cytometry (data not shown). Cells treated with vehicle control (DMSO) showed normal 2N and 4N peaks. Western blotting analysis indicated that CIB1 protein expression amplified in a time dependent manner upon PMA treatment (figure 1A). Quantitation of the band intensity of CIB1 indicated a significant increase in CIB1 expression from day 3 onwards (figure 1B). This suggests that CIB1 protein expression may be elevated in megakaryocytes undergoing endomitosis. To conclusively determine if the increase in CIB1 protein expression observed in Dami cells treated with PMA has physiological merit, we analyzed Cib1 expression in mouse mononuclear bone marrow cells as well as mouse megakaryocytes. Mononuclear bone marrow cells from Cib1 deficient mice were used to validate the specificity of the antibody. In agreement with our Dami cell data, we noticed that Cib1 protein expression was elevated in the megakaryocytes compared to the mononuclear cells (figure 1C).

### Overexpression of CIB1 hinders cell proliferation

**Figure 2:**
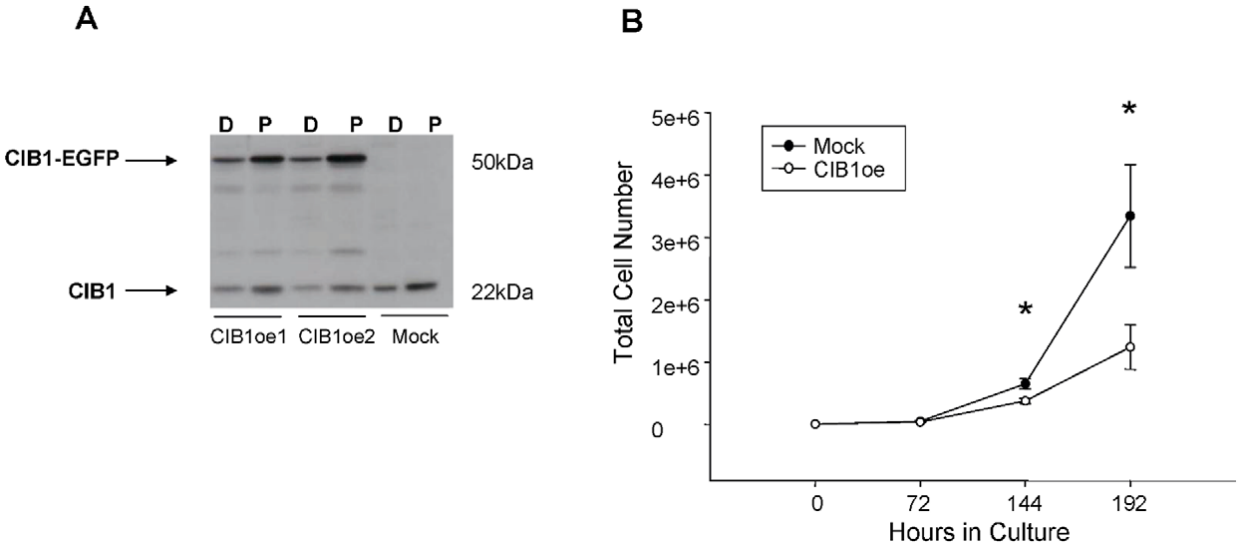
CIB1 overexpression causes reduced cell proliferation. A) Western blot showing endogenous CIB1 as well as CIB1-EGFP expression following selection of stable clones and subsequent PMA treatment for 5 days. D = DMSO; P = PMA. B) Growth curve of CIB1oe and mock Dami cells. Data represent means ± SEM of 6 independent experiments. *P<0.05.

To determine if the increased expression of CIB1 is responsible for observed polyploidy, we stably overexpressed CIB1-EGFP in Dami cells. The CIB1-EGFP band appears at ∼50 kDa in CIB1oe cells, while the CIB1-EGFP band is absent in mock controls (figure 2A). Interestingly, in addition to the endogenous CIB1 band the CIB1-EGFP band is also enhanced upon treatment with 100 nM PMA. This is not surprising considering PMA causes endomitosis in Dami cells, resulting in polyploidy, which will result in increased copy number of the CIB1-EGFP construct.

To evaluate the effect of CIB1 overexpression on the Dami cells, both cell viability and cell proliferation assays were performed. There were no differences in cell viability observed between mock and CIB1oe cells (data not shown). However, cell proliferation was greatly reduced in CIB1oe cells compared to mock (figure 2B). Given the lack of cell death, this suggested that Dami cells overexpressing CIB1 may have a prolonged cell cycle.

### Overexpression of CIB1 impairs the late stages of cell division

**Figure 3:**
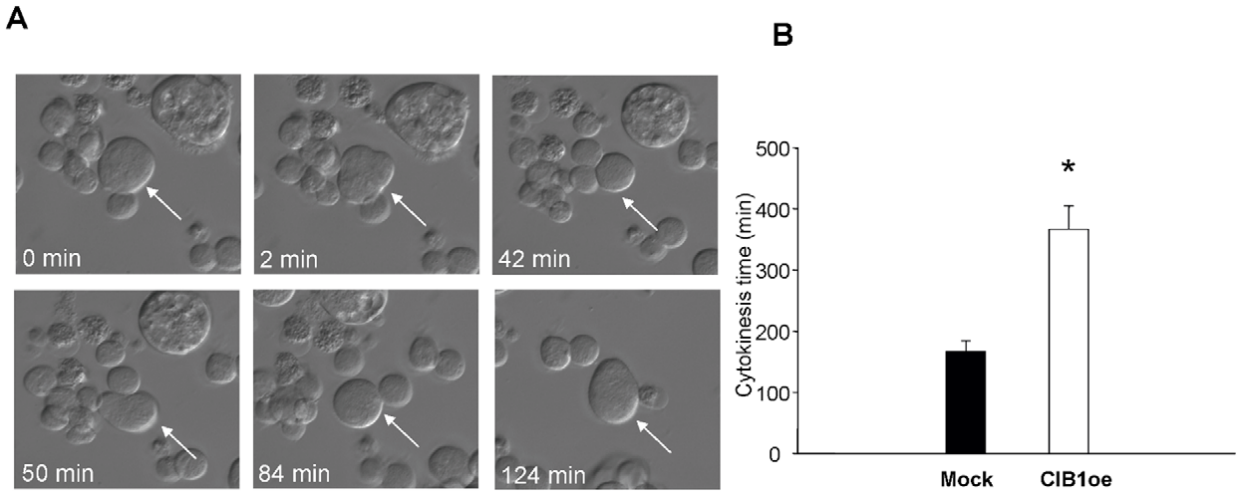
CIB1 overexpression results in delayed cytokinesis. A) Time-lapse microscopy of a CIB1oe Dami cell undergoing furrow ingression followed by furrow regression. B) The time for mock and CIB1oe cells to complete cytokinesis (from furrow formation to division) was quantified using time-lapse microscopy. *p<0.05.

In order to further characterize this observation, we performed time-lapse microscopy of mock and CIB1oe cells. Each cell that developed a furrow was observed and the time it took that cell to complete cytokinesis was recorded. It was also recorded if the cell failed to divide due to furrow regression following furrow formation, as observed in megakaryocytes [2], [3]. An example of one such cell that undergoes endomitosis is shown in figure 3A. A time-lapse movie of the cell indicated by an arrow in figure 3A is included in the supplemental data available online (Movie S1). Interestingly, more CIB1oe (12.5%) cells formed furrows that later regressed as compared to mock (2%). In addition to an increase in furrow regression, CIB1oe cells required nearly twice as long to complete cytokinesis as compared to mock (figure 3B). This was a consistent finding as 44.5% of all CIB1oe cells took over 5 hours to complete cytokinesis compared to only 11.5% of mock. These data suggest that cytokinesis is perturbed upon CIB1 overexpression.

### Overexpression of CIB1 enhances PMA-induced ploidy while depletion of CIB1 inhibits PMA-induced ploidy

**Figure 4:**
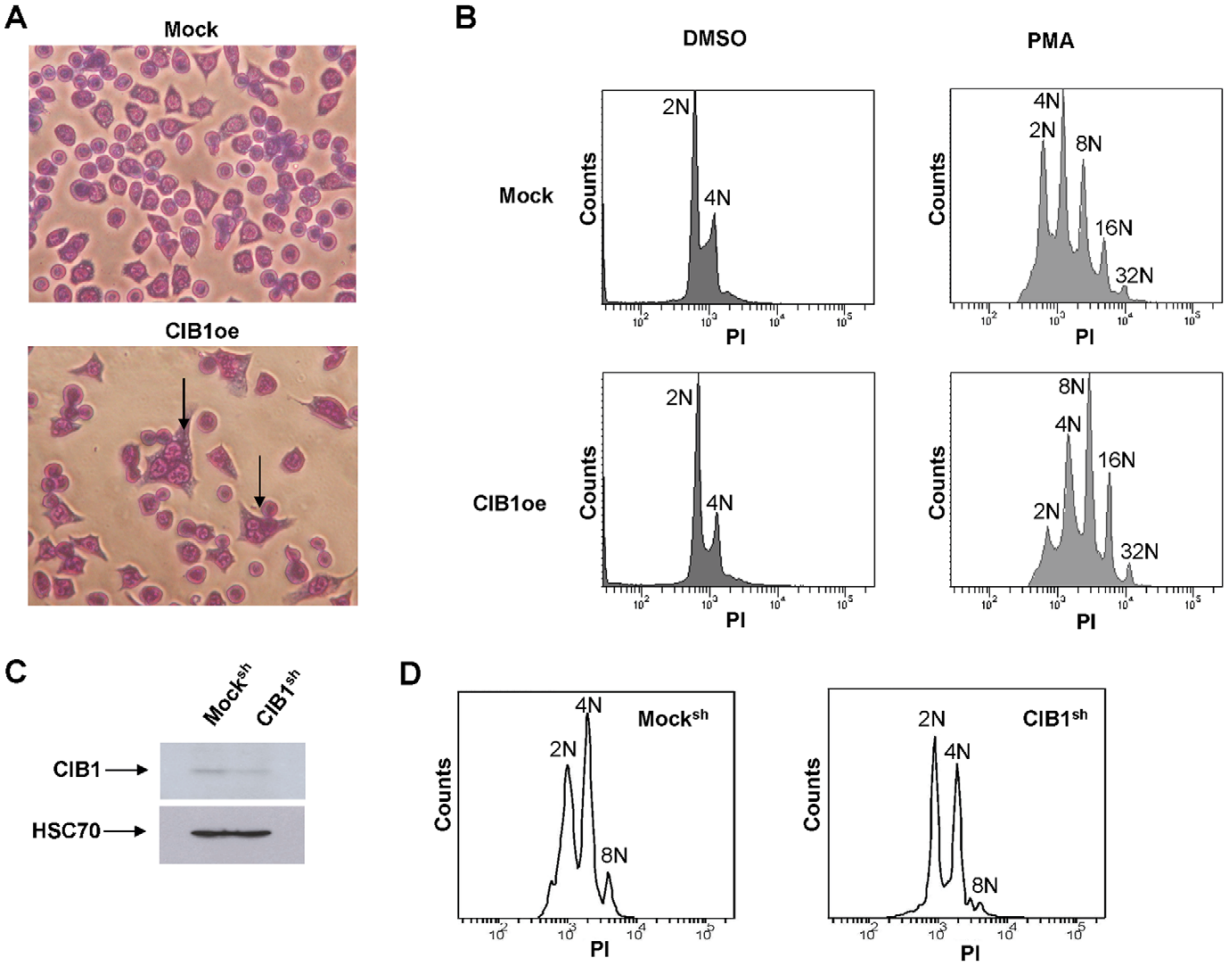
CIB1 augments PMA-induced ploidy in Dami cells. A) Unstimulated CIB1oe and mock cells stained with Wright-Giemsa (arrows indicate multilobated nuclei). B) Representative flow cytometric histograms of DNA content of PMA and vehicle treated mock and CIB1oe cells. C) Western blot of lysates from Dami cells transfected with either CIB1 shRNA (CIB1^sh^) or empty vector (Mock^sh^) and probed with CIB1 and HSC-70. D) Representative flow cytometic histograms of DNA content of 3 day PMA treated CIB1^sh^ and Mock^sh^ cells.

In order to further characterize the morphology of CIB1oe cells, unstimulated cells were allowed to spread on immobilized fibronectin and stained with Wright-Giemsa. Interestingly, we observed what appeared to be a multinucleated phenotype in the CIB1oe cells, which was absent in the mock cells (Figure 4A). However, flow cytometric analysis indicated normal diploid DNA content in unstimulated mock and CIB1oe cells. Therefore, it is possible that CIB1 overexpression induced multilobation of the nucleus, which looked like multinucleation [33]. Interestingly, PMA treatment augmented the extent of polyploidy in CIB1oe cells compared to mock. Shown in Figure 4B are cells treated with PMA for 5 days. In particular, the 2N and 4N populations, which are prominent in mock, are greatly reduced in the CIB1oe cells. In contrast, CIB1oe cells contain substantially higher 8N and 16N populations compared to mock cells (Figure 4B). PMA-dependent increases in CIB1 expression (figure 1) and enhanced polyploidy upon PMA treatment of CIB1 overexpressing cells (figure 4B) collectively suggests that increased levels of CIB1 assist endomitosis. To verify this finding, we depleted CIB1 expression in Dami cells using a CIB1 specific shRNA. We were able to effectively reduce CIB1 expression compared to mock (Figure 4C). Due to the instability of transient transfection only 3 days PMA treatment was performed. Following PMA treatment for 3 days and subsequent flow cytometric analysis, we observed a heightened 4N peak as well as the presence of an 8N and 16N peak in Mock^sh^ cells. However, following the same treatment, CIB1^sh^ cells contained 2N, 4N, and very few 8N cells while no highly polyploid cells were present. This further suggests that CIB1 is an important mediator of endomitosis.

### Overexpression of CIB1 augments surface expression of integrin α_IIb_β_3_

**Figure 5:**
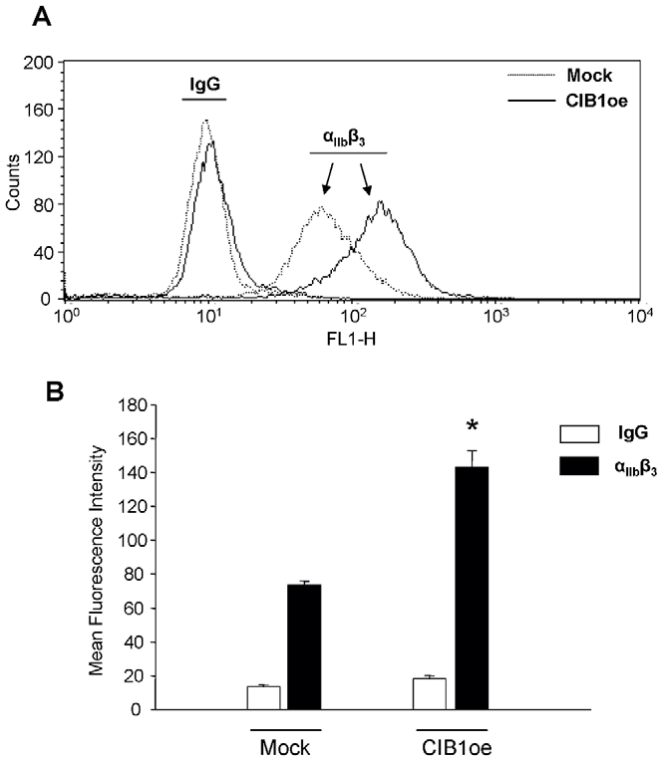
Overexpression of CIB1 enhances surface expression of integrin α_IIb_β_3_. A) Flow cytometric analysis of mock and CIB1oe cells labeled with anti-α_IIb_β_3_ or an isotype matched IgG, followed by a FITC-conjugated secondary antibody. B) Quantification of mean fluorescent intensity derived from (A). The data represent mean ± SEM of 5 independent experiments. *p<0.05 vs mock.

Another characteristic of megakaryocyte differentiation is increased surface expression of integrin α_IIb_β_3_ [34]. Therefore, we next examined the surface expression of α_IIb_β_3_ in CIB1oe and mock cells. Interestingly, CIB1oe cells had enhanced surface expression of α_IIb_β_3_ as compared to mock cells (figure 5A–B). This suggests that CIB1 is responsible for the accumulation of α_IIb_β_3_ at the cell surface during megakaryocyte differentiation. Unlike its role in endomitosis, CIB1 does not require an external cue to produce this effect.

### Interaction of CIB1 with the mitotic kinase Plk3 is amplified upon PMA treatment

**Figure 6:**
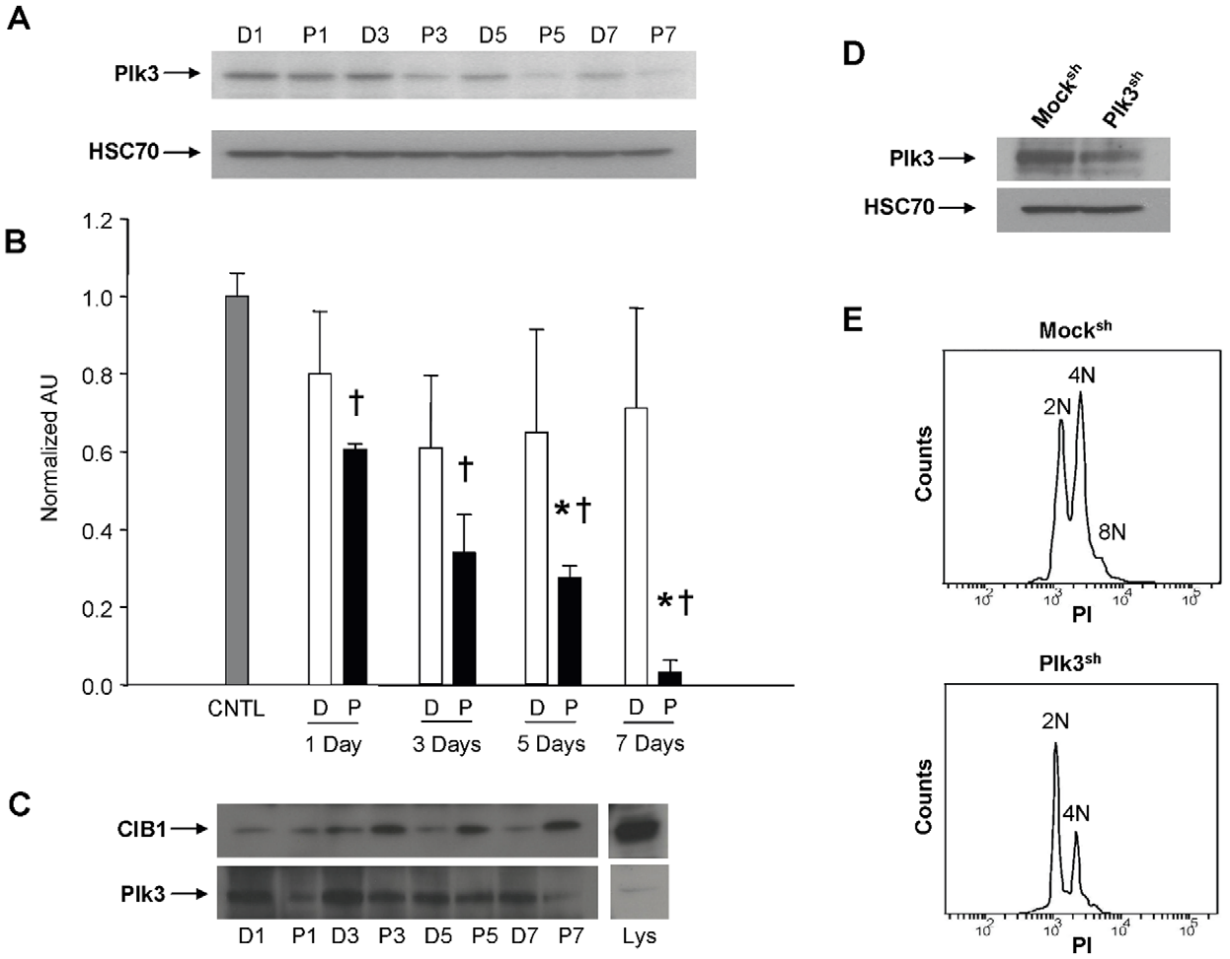
Plk3 protein expression is reduced while its interaction with CIB1 is enhanced upon PMA treatment. A) Western blots showing Plk3 protein expression in response to PMA treatment of Dami cells. Equal loading was assessed using HSC-70. D = DMSO as vehicle control; P = PMA; *p<0.05 vs mock. B) Densitometric data from (A) quantified and normalized to untreated control (CNTL) samples. †p<0.05 compared to CNTL; *p<0.05 compared to corresponding DMSO value. C) Representative Western blots of co-immunoprecipitation of CIB1 with Plk3 from Dami cell lysates. The blot was initially probed for CIB1, then stripped and reprobed for Plk3. Lys = Lysate, showing input. D) Western blot of lysates from Dami cells transfected with either Plk3 shRNA (Plk3^sh^) or empty vector (Mock^sh^) and probed for Plk3 and HSC-70. E) Representative flow cytometic histograms of DNA content of 3 day PMA treated Plk3^sh^ and Mock^sh^ cells.

To further understand the mechanism by which CIB1 affects endomitosis, we examined the expression of a recognized binding partner of CIB1, Plk3, which is known to regulate the cell cycle [8]. Interestingly, and in direct contrast to the expression of CIB1, we found that Plk3 protein expression decreased upon PMA treatment in a time-dependent manner (figure 6A–B). We next sought to determine if CIB1 interacted with Plk3 in polyploid cells. We observed that CIB1 interacted with Plk3 in both vehicle and PMA treated Dami cells (figure 6C). However, the interaction increased with the length of PMA treatment, suggesting that heightened CIB1/Plk3 interaction correlates with increased DNA content, even though Plk3 protein expression decreases. In order to determine if endomitosis is dependent upon reduced Plk3 protein expression, we depleted Plk3 expression by ∼40% using a Plk3-specific shRNA (Figure 6D). PMA treatment (100 nM for 3 days) of Mock^sh^ cells induced endomitosis. Surprisingly, endomitosis was not observed in similarly treated Plk3^sh^ cells (Figure 6E). PMA treated Mock^sh^ cells had a prominent 4N peak and an 8N peak, while PMA treated Plk3^sh^ cells had a prominent 2N peak and a small 4N peak. Interestingly, we recently revealed that Plk3 is necessary to localize CIB1 to the MTOC [22].

The preceding data suggest that, during endomitosis, CIB1 protein expression is increased, while Plk3 protein expression is decreased. Furthermore, CIB1 interacts with Plk3 and that interaction is heightened with prolonged PMA treatment. Additionally, knockdown of CIB1 expression suppresses endomitosis. Similarly, reduction of Plk3 expression inhibited endomitosis. This appears contradictory because Plk3 protein expression decreases with increasing polyploidy. However, like CIB1, Plk3 regulates multiple cellular processes some of which may be necessary during endomitosis. Because Plk3 depletion inhibited PMA-induced polyploidization, it is likely that Plk3 expression is necessary for the onset of endomitosis.

## Discussion

In this report, we demonstrate that CIB1 is involved in the regulation of endomitosis. We found that, following PMA treatment of Dami cells, CIB1 protein expression increases, that CIB1 and Plk3 interact, and that this interaction was enhanced with prolonged PMA treatment, even though Plk3 protein expression decreased. Furthermore, overexpression of CIB1 in Dami cells enhanced the megakaryocyte phenotype as characterized by defective cytokinesis, heightened α_IIb_β_3_ surface expression, and increased DNA content following PMA exposure.

CIB1 interacts with a variety of proteins and regulates a number of cellular processes [8], [17], [18], [19]. One such protein is Plk3 [8]. Furthermore, we have recently demonstrated that CIB1 inhibits the kinase activity of Plk3 in a calcium-dependent manner [21], [22]. It was previously reported that overexpression of Plk3 had an adverse effect on cytokinesis [35]. Additionally, Wang et al. demonstrated that Plk3 localizes to the spindle poles and mitotic spindles, as well as to the midbody during metaphase and telophase, respectively [9]. Furthermore, manipulation of Plk3 had a profound effect on microtubule dynamics [9]. As reported here we observed a reduction in Plk3 protein expression as well as a heightened CIB1/Plk3 interaction, which corresponded to the increase in DNA content in Dami cells. Additionally, RNAi-mediated knockdown of CIB1 led to reduced PMA-induced ploidy. We have previously shown that CIB1 inhibits Plk3 activity [21]. Therefore, we hypothesized that knockdown of Plk3 would enhance PMA-induced ploidy. Interestingly, Plk3 knockdown showed reduced ploidy. This is not surprising, because, we have previously reported that Plk3 is responsible for localizing CIB1 to the MTOC where it may serve to regulate microtubule dynamics [21], [22]. Therefore, it is possible that Plk3 is necessary during early rounds of endomitosis, perhaps to localize CIB1 to the MTOC. However, it is also plausible that Plk3 is dispensible for endomitosis. In fact, Huang et al., demonstrated that Plk3 protein expression actually increased during endomitosis in K562 cells [36]. Clearly, further characterization of the spatio-temporal dynamics of Plk3 in endomitotic cells is required to conclusively determine if Plk3 is an important mediator of endomitosis.

Both CIB1 and Plk3 are expressed in myriad cell types. Additionally, both CIB1 and Plk3 interact with a variety of other proteins and participate in numerous cell functions [11], [17], [18], [19], [37], [38], [39]. It is likely that CIB1 and Plk3 regulate several distinct cell functions through altered expression level and/or altered subcellular localization. Endomitosis occurs only in very specialized cell types such as a megakaryocyte. Therefore, it is notable that expression of both CIB1 (upregulated) and Plk3 (downregulated) is altered during this process.

Our finding that overexpression of CIB1 in Dami cells caused increased incidence of furrow regression is consistent with a phenotype observed in early megakaryocytes [3], [40], [41]. The mechanism leading to furrow regression remains unclear as several likely candidate proteins, those of the chromosomal passenger complex, appear to function normally in megakaryocytes [42], [43]. Interestingly, it was recently revealed that Plk3 interacts with members of the chromosomal passenger complex [44]. It is possible that CIB1 may interact with, and regulate an additional key protein involved in cytokinesis. One such candidate protein that interacts with CIB1 and may have an impact on cytokinesis is p21 Activated Protein Kinase-1 (PAK1) [19]. When active, PAK1 is able to phosphorylate and activate Lin-11/lsl-1/Mec-3 kinase 1 (LIMK1), which in turn inhibits cofilin by phosphorylation [19], [45]. Furthermore, cofilin activity is required for contractile ring formation and maintenance during cytokinesis [46], [47]. Therefore, it is possible that increased CIB1 protein expression during PMA treatment of Dami cells, or during megakaryocyte differentiation, allows CIB1 to bind and activate PAK1 thus inhibiting cofilin phosphorylation and perturbing contractile ring integrity. This possibility can not be ruled out at this time and further studies are needed to determine if CIB1 associates with PAK1 during megakaryocyte differentiation, and whether or not that association has any effect on contractile ring function.

Both CIB1 and Plk3 knockout mice have been produced [10], [26]. *Cib1^-/-^* mice have a defective thrombosis due to impaired integrin α_IIb_β_3_ dependent outside-in signaling [29]. However, megakaryopoiesis has not been characterized in these mice. It would be interesting to determine if megakaryocyte maturation is inhibited in the absence of CIB1, but enhanced in the absence of Plk3.

## Supporting Information

Movie S1 CIB1 overexpression results in delayed cytokinesis. Time-lapse video of CIB1 overexpressing Dami cells undergoing furrow regression.
(4.88 MB MOV)
